# Truth and Transparency in Crisis Pregnancy Centers

**DOI:** 10.1089/whr.2020.0057

**Published:** 2020-07-27

**Authors:** Carly Polcyn, Sarah Swiezy, Leah Genn, Pavithra Wickramage, Neha Sidiqqui, Candise Johnson, Pooja Nair, Caitlin Bernard, Velvet Miller

**Affiliations:** ^1^ University of Toledo—Health Science Campus, Toledo, Ohio, USA.; ^2^ Indiana University School of Medicine, Indianapolis, Indiana, USA.; ^3^ Department of Obstetrics and Gynecology, Indiana University School of Medicine, Indianapolis, Indiana, USA.; ^4^ Florida State University, Tallahassee, Florida, USA.; ^5^ UNTHSC, Fort Worth, Texas, USA.; ^6^ Carle Illinois College of Medicine, Champaign, Illinois, USA.; ^7^ University of Mississippi Medical Center, Jackson, Mississippi, USA.

**Keywords:** women in medicine, crisis pregnancy centers, pregnancy counseling centers

## Abstract

The prevalence of crisis pregnancy centers (CPCs), their false claims, and the real harm they cause necessitate public education about their unethical practices. Also called “pregnancy resource centers” and “pregnancy support centers,” CPCs are nonmedical institutions designed to deceive women seeking comprehensive pregnancy care, as their volunteers are instructed to pedal misinformation about reproductive health care.

Dr. Caitlin Bernard, an abortion provider in Indianapolis, has seen patients seeking an abortion who were deceived by crisis pregnancy centers (CPCs) posing as health care providers, setting up shop next door to legitimate medical centers. Instead of getting necessary care at CPCs, women instead receive misinformation, which serves only to confuse and delay their care. One patient explained her experience to Dr. Bernard: “They told her ‘Come back and do another ultrasound so that you can see the baby moving. Then it may change your mind.’ She felt like she couldn't trust what they were saying, that they clearly had an agenda in mind because they were trying to dissuade her from what she wanted. And when she came to us, of course, she was further along and was no longer eligible for a medication abortion and had to have a surgical abortion.”

Dr. Bernard stated that this is not an unusual scenario in her practice. CPCs are pervasive in the reproductive health landscape. CPCs have been quietly thriving for years, whereas planned parenthood and abortion providers have faced constant scrutiny and restrictive laws since Roe v. Wade. Their ulterior motives, lack of qualifications, and disreputable services are being used to misinform women, both in Indiana and around the country. For example, a study found that CPCs were claiming there was a 25%–30% chance of having a spontaneous miscarriage in a pregnancy, implying that there was a high likelihood there would be no need to get an abortion.^1^

The Women in Medicine Committee of the American Medical Association (AMA)-Medical Student Section conducted a poll revealing that 15% of medical students “have no knowledge regarding CPCs,” another 17% “are familiar with the term CPC but do not know much beyond that,” and 47% “know some details about CPCs.”^2^ Only 21% endorsed the statement: “I have a comprehensive understanding about CPCs.”^2^ The medical students who were polled are a self-selected group, active within the AMA, and highly motivated to keep up with policy and current political issues.^2^ This is to say, these are the students who arguably have the highest likelihood of having knowledge about CPCs, and yet, they are still in the dark.

There is a dire need to widely disperse information about CPCs to medical students, other health professionals, and the general public. We are taking the first step by summarizing the literature, evidence, and issues into the categories hereunder.

## Prevalence of CPCs as Compared with Licensed Medical Abortion Providers

As of 2019, there are 2537 CPCs across the United States. The vast majority are operated by one of two major evangelical religious organizations: Care Net International and Heartbeat International.^3^ In contrast, there are 780 clinics providing abortion services in the country.^4^ In the most extreme case, Missouri has one single abortion facility and 69 CPCs.

## Messaging and Advertising

In a study examining 254 websites representing 348 CPCs, 80% of them were found to provide at least one false or misleading piece of information.^1^ For example, CPCs strategically place advertisements aimed at pregnant women on search engine results for abortion-related terms. Their ads strive to display the appearance of abortion-providing medical clinics and are frequently placed on billboards and buses near abortion clinics.^5^ In addition, CPCs often intentionally occupy buildings near abortion-providing clinics,^5^ as in the case of the clinic where Dr. Bernard works in Indianapolis. CPCs have also developed initiatives specifically targeting communities of color, a population that faces significant barriers, such as financial inequity, shortage of health care providers, and lack of health insurance. By building centers in these areas, CPCs present themselves as often the only available option for reproductive health services.^5^

## Services and Personnel

CPCs are intentionally advertised as comprehensive medical facilities with licensed clinical professionals despite offering only select services and being largely staffed by volunteers. CPCs, as nonmedical entities, are not held to the same inspection, safety, and regulation requirements as medical facilities. In fact, CPCs have no such requirements at all.^1^ Inside CPCs, staff often use manipulative and coercive tactics on unsuspecting women: some volunteers wear white coats despite having no medical training, they fail to disclose that they are not a medical facility, and they express judgment toward clients about their decisions to pursue abortion or contraception. They offer ultrasound services, which they may not be licensed to interpret, for the purpose of using fetal images to deter women from abortion. They quote falsehoods linking abortion to adverse mental health sequelae, breast cancer, and future infertility.^6^

## Funding

CPCs publicly pose as nonprofit organizations, but many utilize public tax dollars to provide their services. One of the primary means of funding is through diversion of funds from Temporary Assistance for Needy Families (TANF). According to ThinkProgress, in 2016 $1.7 million in TANF funds were given to CPCs in Indiana alone.^7^ The justification given for siphoning these funds meant to support families living in poverty included the following: “(1) Encouraging the formation and maintenance of two-parent families while providing pregnancy support services to expectant parents; and (2) Preventing and reducing the incidence of adolescent and out-of-wedlock pregnancies.”^7^

Although it is possible that the rates of unintended pregnancy can potentially be decreased by CPCs, CPCs do not provide the comprehensive care that has been evidence backed and shown to reduce pregnancies and abortions: they do not provide comprehensive contraception services.^8^ Furthermore, there has been no formal studies of the efficacy of CPCs in reducing abortion rates. However, comprehensive care has been shown to be significantly effective at reducing abortion incidence. For example, in Colorado, after the state expanded access to long-acting reversible contraceptives—a service not offered by CPCs—the teen abortion rate decreased 40% between 2009 and 2014.^9^ This rejection of evidence-based medicine is counterintuitive to the stated goal of CPCs to reduce abortion rates and is in direct contradiction to the justification given for using public monies to fund the operation of CPCs. This leads to the question: why is TANF funding being siphoned to CPCs at all?

Since CPCs receive a large amount of money, they are able to continue providing free services. This makes these centers attractive for low-income, uninsured, and/or undocumented women. These nonmedical facilities are sometimes seemingly their only option for receiving information, be it false or not. We also know that, in the United States, a majority of unintended pregnancies occur in these same vulnerable populations of low income and minority women, which makes the work of CPCs all-the-more exploitative.

Many organizations have brought attention to the detrimental practices upheld by CPCs. The Society for Adolescent Health and Medicine as well as the North American Society for Pediatric and Adolescent Gynecology both state that CPCs pose substantial risk to women by failing to adhere to medical and ethical practice standards.^10^ The American College of Obstetricians and Gynecologists, the AMA, and many other medical societies support access to comprehensive reproductive health services—which, again, are not provided by CPCs.^11^

The issue of access to safe reproductive health care cannot be settled with deceit. Misinformation is not the answer. Our patient in Indiana was able to find proper care despite her experience, but others are not so fortunate. We must shed light on the intentionally fraudulent practices of CPCs, because women deserve better.

## Abbreviations Used

AMA: American Medical Association
CPC: crisis pregnancy center
TANF: Temporary Assistance for Needy Families
